# The Interplay between Airway Cilia and Coronavirus Infection, Implications for Prevention and Control of Airway Viral Infections

**DOI:** 10.3390/cells13161353

**Published:** 2024-08-14

**Authors:** Xuyao Dai, Ruodan Xu, Ning Li

**Affiliations:** Department of Biomedical Engineering and Technology, Institute of Basic Theory for Chinese Medicine, China Academy of Chinese Medical Sciences, Beijing 100700, China; daixuyao000@163.com

**Keywords:** ciliogenesis, cilia, coronavirus, respiratory diseases

## Abstract

Coronaviruses (CoVs) are a class of respiratory viruses with the potential to cause severe respiratory diseases by infecting cells of the upper respiratory tract, bronchial epithelium, and lung. The airway cilia are distributed on the surface of respiratory epithelial cells, forming the first point of contact between the host and the inhaled coronaviruses. The function of the airway cilia is to oscillate and sense, thereby defending against and removing pathogens to maintain the cleanliness and patency of the respiratory tract. Following infection of the respiratory tract, coronaviruses exploit the cilia to invade and replicate in epithelial cells while also damaging the cilia to facilitate the spread and exacerbation of respiratory diseases. It is therefore imperative to investigate the interactions between coronaviruses and respiratory cilia, as well as to elucidate the functional mechanism of respiratory cilia following coronavirus invasion, in order to develop effective strategies for the prevention and treatment of respiratory viral infections. This review commences with an overview of the fundamental characteristics of airway cilia, and then, based on the interplay between airway cilia and coronavirus infection, we propose that ciliary protection and restoration may represent potential therapeutic approaches in emerging and re-emerging coronavirus pandemics.

## 1. Introduction

Mucociliary clearance (MCC) is a key self-protection mechanism in the human respiratory tract that functions to remove foreign pathogens from the airways. The function of the MCC depends primarily on the ciliated epithelial cells (CECs), as well as the mucus layer, which traps foreign pathogens, and a low-viscosity periciliary layer (PCL), which lubricates airway surfaces and facilitates ciliary beating for efficient mucus clearance [1]. As one of the essential components of the respiratory MCC, cilia beat in metachronous waves to expel pathogens and inhaled particles trapped in the mucus layer from the airways, thus playing an important role in defending the respiratory system against the invasion of pathogens and pollutants [2].

Coronaviruses (CoVs) are the major cause of respiratory infections. Seven known CoVs, namely HCoV-229E, HCoV-OC43, HCoV-NL63, HCoV-HKU1, SARS-CoV, MERS-CoV, and SARS-CoV-2, have been identified as human pathogens, with the latter three being highly pathogenic and capable of causing severe respiratory disease and even life-threatening illness [3]. The binding receptors for different coronaviruses can vary. For example, MERS-CoV uses dipeptidyl peptidase 4 (DPP4), SARS-CoV employs angiotensin converting enzyme 2 (ACE2), and SARS-CoV-2 utilizes both ACE2 and transmembrane protease serine 2 (TMPRSS2). Both ACE2 and TEMPRSS2 have been observed to localize extensively to the ciliary membranes of the respiratory tract [4]. Upon viral entry into the airways, the virus targets cilia or epithelial cells, leading to the disruption of ciliary motility and ultrastructure, and the dysfunction of mucociliary clearance, thereby facilitating host infection [5,6]. Concomitantly, the expression of cilia-associated cytoskeletal proteins undergoes substantial alterations. For instance, the ciliary structural proteins dynein axonemal heavy chain 7 (DNAH7), dynein axonemal intermediate chain 2 (DNAI2), and intraflagellar transport 27 (IFT27) have been demonstrated to exhibit a significant reduction in expression levels [5]. In particular, the intraflagellar transport (IFT) family has been shown to possess immunomodulatory functions, and the downregulation of IFT88 can affect the NF-κB signaling pathway and the expression of several downstream inflammatory cytokines [7]. In addition, upon viral entry into the respiratory tract, ciliated sensory receptors, including bitter taste receptors (T2Rs) and transient receptor potential (TRP) channels, also respond. Of these, taste 2 receptor member 38 (TAS2R38) has been associated with the severity of SARS-CoV-2 infection, while transient receptor potential vanilloid 4 (TRPV4) is acutely activated by the receptor-binding domain (RBD) of the SARS-CoV-2 spike (S) protein, promoting apoptosis [8,9].

In conclusion, respiratory cilia fulfill a multitude of functions. In addition to their role in mucociliary clearance, airway cilia also engage in immunoregulatory and chemosensory activities. However, these functions of the cilia are frequently overlooked when investigating the prevention and treatment of respiratory viruses. Despite the fact that cilia represent the first point of contact between the respiratory tract and coronaviruses, there is still a need to gain a deeper understanding of how ciliary function and expression are altered during viral infection, as well as which cellular pathways are involved. This review will provide advanced insight into the dynamic relationship between cilia and coronaviruses, with the aim of elucidating the functions and regulatory mechanisms of airway cilia during coronavirus infection.

## 2. Airway Cilia

### 2.1. Structure and Distribution of Airway Cilia

The epithelial surface of the respiratory tract is exposed to dust and pathogens on a daily basis, which presents a significant challenge to the respiratory system. Normal MCC plays a pivotal role in the prevention of damage caused by dust and pathogens and in the maintenance of respiratory defenses. The respiratory MCC consists of a motor system (cilia), a mucus blanket (mucus and periciliary layer), and mucus-secreting cells (goblet cells, serous cells, and secretory glands) [10].

**Figure 1:**
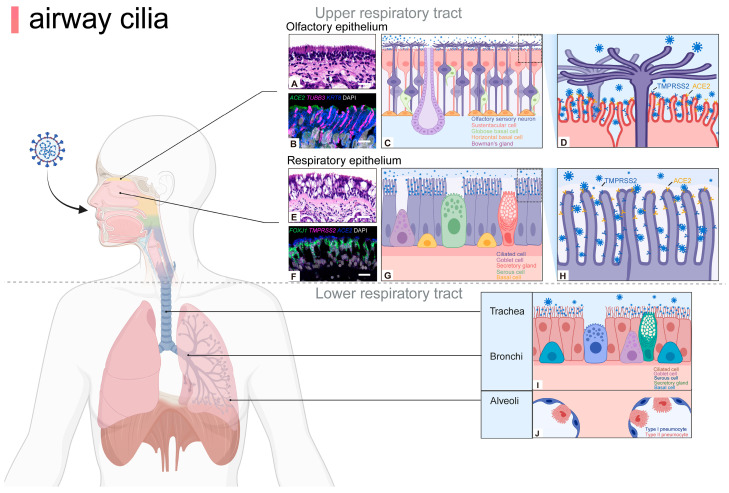
Airway cilia are distributed on the apical epithelial surface of upper and lower respiratory tracts. The nasal epithelium is divided into a RE and OE, whose functions and cell types differ. In the OE, cilia extend over the dendrites of mature olfactory neurons and are responsible for odor detection, whereas ciliated cells in the RE mainly play a role in cleaning pathogens. Moreover, the coronavirus-binding receptors ACE2 and TMPRSS2 are markedly expressed on the cilia of respiratory epithelial cells and the microvilli of olfactory epithelial sustentacular cells, though not specifically localized on olfactory cilia (**A**–**H**). Brightfield images of hematoxylin and eosin-stained sections (**A**,**E**), confocal images of sections stained fluorescently with RNAscope and IHC (**B**,**F**) [18]. Thus, cilia at different sites may play different roles during viral infection. In the lower airways, ciliated cells are distributed in the epithelium of the trachea and bronchi for pathogen clearance (**I**,**J**). ACE2, angiotensin converting enzyme 2; FOXJ1, forkhead box protein J 1; KRT8, keratin 8; OE, olfactory epithelium; RE, respiratory epithelium; TMPRSS2, transmembrane protease serine 2; TUBB3, tubulin beta 3 class III. Scale bars: 20 µm (**A**,**F**), 10 µm (**B**), 50 µm (**E**). Created with BioRender.com.

**Figure 2:**
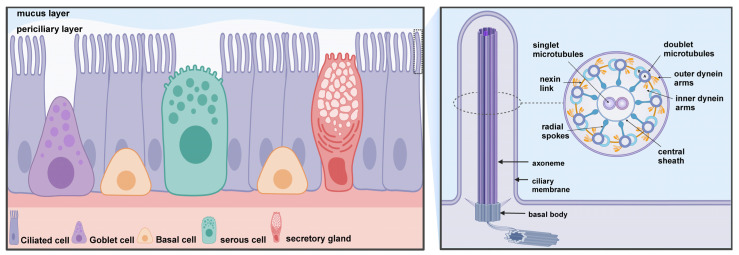
Mucociliary clearance (MCC) consists of two primary components: motile cilia and mucus blanket (mucus and periciliary layer). MCC is a critical defense of the airways and is dependent on a highly coordinated ciliary function. Ciliary kinetic function is dependent on the special structure of the cilia. The core structure of the cilium is a highly conserved “9 + 2” axoneme that extends from the basal body in the apical region of ciliated cells into the periciliary layer, with the cilia tips reaching the mucus layer of the airway lumen. The ciliary axoneme consists of 9 groups of microtubule doublets and a pair of central single microtubules. Cilia contain a variety of protein complexes, including dynein arms, nexin link, radial spokes, and central sheath that connect the microtubules to each other. Created with BioRender.com.

As a crucial component of the MCC, ciliated cells exhibit ubiquitous distribution along the entire respiratory epithelium, from the nasal cavities, sinuses, and pharynx to the epithelium of the trachea and bronchi (Figure 1). Cilia are morphologically tiny protrusions on the surface of cells, measuring 5 to 10 μm in length and approximately 0.2 μm in diameter. Each cell exhibits a cilia count of approximately 200–300, in addition to a considerable number of microvilli that extend from the respiratory epithelium [11]. The cilia are encapsulated by the cytoplasmic membrane and contain a basal body, axoneme, and ciliary matrix within their interior (Figure 2). The basal body represents the structure situated at the base of the cilium, serving as the anchor point for the cilium and playing a critical role in the formation and maintenance of the cilium. The axoneme constitutes a microtubular skeleton within the cilium. Cilia can be classified according to their axial structure, which distinguishes between motile cilia (“9 + 2” structure) and immotile cilia (“9 + 0” structure) [12]. The respiratory cilia follow the “9 + 2” pattern, comprising nine groups of microtubule doublets and a pair of single microtubules in the center. Each peripheral microtubule doublet is connected by a complete microtubule A and a “C”-shaped microtubule B, and is connected by a nexin-dynein regulatory complex (N-DRC), with microtubule A extending a radial spoke to connect the outer microtubule doublet to the central microtubule [13]. Meanwhile, two force arms, the outer dynein arm and the inner dynein arm, extended outward from microtubule A to adjacent microtubules B. These are bridging molecules between microtubules that allow adjacent microtubules to slide relative to each other, thereby providing the force for cilia oscillation. Moreover, the central microtubule, the radial spoke, and the N-DRC enable the regulation of the oscillatory form [14]. Adenosine triphosphate (ATP) is a major source of energy to support the ciliary beat by facilitating relative sliding between microtubule dimers, thereby generating anteroposterior propulsion and causing the cilia to bend [15]. This suggests that the coordinated action of microtubules, ATP-driven dynein motors, and regulatory complexes generates ciliary movement.

Within the upper respiratory tract, the nasal epithelium is composed of two distinct types of nasal epithelium: respiratory epithelium (RE) and olfactory epithelium (OE), both of which contain basal cells (Figure 1). Basal cells can be divided into two types: horizontal basal cells (HBCs) and globular basal cells (GBCs). HBCs specifically express keratin 5/6/14 (KRT5/6/14), intercellular adhesion molecule 1 (ICAM1), paired box 6 (PAX6), and SRY-box transcription factor 2 (SOX2); while GBCs express SOX2 and mammalian achaete-scute homologue 1 (MASH1) [16]. Typically for the respiratory mucosa, ciliated cells express forkhead box protein J 1 (FOXJ1), epithelial cells express epithelial cell adhesion molecule (EPCAM), and the mucin-5AC (MUC5AC) labels goblet cells; while in the olfactory mucosa, olfactory marker protein (OMP) and tubulin beta 3 class III (TUBB3) are markers for mature olfactory neurons whose dendrites form cilia that specifically express acetyl-alpha tubulin and adenylate cyclase 3 (ADCY3), and sustentacular cells express KRT8, secretory cells specifically express BPI fold containing family A member 1 (BPIFA1) [17,18]. Despite the presence of cilia with a “9 + 2” structure in the olfactory sensory neurons (OSN) of the OE, the absence of dynein arms, which are necessary for movement, renders them incapable of movement. Consequently, their function is limited to odor detection through the interaction of receptors on dendritic cilia [19]. In the lower respiratory tract, the proportion of ciliated cells increases with the number of airway branches, from 47 ± 2% of the tracheal epithelium to 73 ± 1% of the small airway epithelium [20]. However, with the formation of the succeeding airways in the peripheral lungs, the length of the cilia gradually shortens [21].

Airway cilia have components typical of motile cilia. To investigate the structure and regulation of motile cilia, *Chlamydomonas* and *Paramecium* are often chosen as analogous models. It has been proven that motile cilia and eukaryotic flagella share the same basic biochemical mechanisms of motion generation and control [22]. Proteins from purified flagella of the green alga *Chlamydomonas reinhardtii* were identified by mass spectrometry, of which 360 proteins were identified with high confidence and a further 292 with moderate confidence [23]. Interestingly, homologues of these *Chlamydomonas* proteins have been identified by human genome sequencing [24]. In addition, proteomic analysis of cilia isolated from human airway epithelial cells in in vitro air-liquid interface (ALI) culture identified over 200 axonemal proteins, some of which are conserved with those found in other motile cilia [24], and a few proteins, such as sperm autoantigenic protein 17 (SPA17), sperm associated antigen 6 (SPAG6), and retinitis pigmentosa protein 1 (RP1), which is associated with photoreceptor axonemal structures, are unique [24]. Furthermore, characterization of the transcriptome of murine tracheal epithelial cells has revealed similarities between components of motile and primary cilia, and many of the genes identified as part of the mouse ciliary cell transcriptome correspond to human airway ciliary proteins, indicating the high degree of conservation of ciliary molecules across species [25].

### 2.2. Development and Function of Airway Cilia

**Figure 3:**
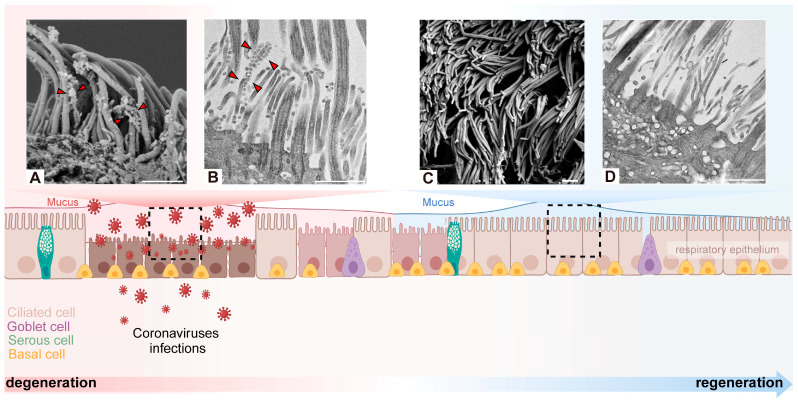
Degeneration and regeneration of cilia after coronavirus infection. Coronavirus binds to the motile cilia during early viral infection. Representative images of virions attaching to motile cilia and causing extensive cilia lodging and axoneme rupture as observed by scanning electron microscope (**A**) or transmission electron microscopy (**B**) [43]. After coronavirus infection causes the loss of cilia, it triggers basal cells to differentiate into ciliated cells in order to maintain the homeostasis of the airway epithelium. Through scanning electron microscope and transmission electron microscope, it was observed that the normal cilia were densely arranged, protruding from the epithelial cells, with a diameter of about 250–500 nm and a length of 5–10 µm (**C**,**D**) [43]. Arrowhead: virus particles. Scale bars: 1 µm (**A**–**D**). Created with BioRender.com.

The development of respiratory cilia is a complex process that involves multiple stages, beginning with the embryonic phase and developing to the adult stage, where cilia undergo continuous renewal. In mice, the movement ciliary system, which is responsible for the clearance of amniotic fluid from the lungs, initiates on the third day of life [26]. The process of human airway ciliogenesis commences in the seventh week of embryonic development and ends prior to birth. Abnormal cilia at this stage have been associated with respiratory distress in newborns [27]. Specific genetic disorders, such as primary ciliary dyskinesia (PCD), also impact the development and functionality of the cilia. In this condition, the cilia are either absent or exhibit structural abnormalities that impede their ability to vibrate effectively, thereby impairing their capacity to clear the airways [28]. Previous studies have shown that airway cilia are mature and terminally differentiated after birth [29]. A defect in airway motile cilia may impair mucociliary clearance during the transition from fetal to neonatal life, leading to atelectasis and lobar collapse and causing neonatal respiratory distress [30]. More than 80% of neonates with primary ciliary dyskinesia develop symptoms within 1–2 days of birth [31]. Moreover, ciliated cells are capable of self-renewal. This indicates that aged cilia are replaced by new ones, a process that depends on the activity of basal stem cells to guarantee the continuity and efficacy of cilia function. Furthermore, damage to the cilia may occur when the respiratory tract is compromised, for instance, as a result of infection or the inhalation of pollutants. In such instances, ciliated cells initiate repair mechanisms with the objective of restoring the normal structure and function of the cilia [32] (Figure 3).

As constituent elements of the respiratory epithelium, cilia perform a variety of vital physiological functions. In addition to the aforementioned functions, airway cilia play a pivotal role in maintaining the function of mucus cilia as an “escalator” to ensure the airway remains unobstructed and clean [33]. Mucus is secreted by goblet cells and by mucous cells in the submucosal glands and is the primary line of defense for trapping particles [34]. The oscillatory movement of the cilia propels the mucus layer in a direction towards the oropharynx, thereby maintaining the cleanliness of the airways [35]. At the apical end of the respiratory cilia, there is a high concentration of mitochondria, which provide the energy necessary for ciliary movement and maintain the ability of the respiratory cilia to move autonomously [36]. Respiratory cilia are capable of oscillating and beating across multiple cells, thereby creating wave-like motions on the epithelial surface [37]. The speed of cilia movement is considerable, with the typical frequency of cilia oscillation being in the range of 10–20 Hz. The movement of cilia can facilitate the replacement of the mucus layer on the mucosal surface at a specific speed, with the mucosal layer being replaced two or three times per hour [38]. Ciliary movement also occurs in a specific direction. The ciliary movement of the upper respiratory tract expels mucus containing dust and pathogens from the nasal cavity into the nasopharynx. In the trachea and bronchi, the direction of ciliary movement is towards the throat, then slightly to the pharynx. Subsequently, the mucus mass is either swallowed or coughed out, resulting in a cleansing effect on the airways [39].

In addition to their function of cleaning the airways, respiratory cilia are also capable of sensing the presence of harmful substances entering the airways. In microarray expression data from primary cultures of differentiated human airway epithelia, the T2R family (such as T2R4, T2R43, T2R38, and T2R46) and T2R pathway-related proteins (such as α-gustducin, phospholipase C-β2 (PLC-β2), and transient receptor potential melastatin 5 (TRPM5) were found to be expressed in ciliated cells and specifically localized to cilia. Following the activation of T2R by the bitter compound denatonium, ciliary activity is stimulated, and the clearance of harmful substances is accelerated [40]. This suggests that respiratory cilia are capable of detecting signals and that the T2R signaling system may also be involved in the infection of respiratory pathogens. For instance, in cystic fibrosis, the lungs are typically infected with *P. aeruginosa*, which produces lactone quorum sensing molecules that can activate certain bitter receptors [41]. The expression of the bitter receptor TAS2R38, which is a modifier gene for cystic fibrosis, has been shown to correlate with the severity of SARS-CoV-2 infection. This suggests that stimulating the expression of TAS2R38 may present a potential therapeutic strategy for the treatment of SARS-CoV-2 infection [9]. In addition, previous studies have indicated that a category of channel proteins that are responsible for sensory responses, namely TRP channel proteins, exhibit high levels of expression in the respiratory epithelium. For instance, TRPV4 is expressed on the ciliary membrane of airway epithelial cells and responds to mechanical loading, shear stress, and osmotic pressure by altering ciliary beat frequency [42]. In conclusion, the primary functions of airway cilia can be summarized as follows: first, they maintain respiratory cleanliness; second, they serve as sensors of the external environment. This enables them to play a crucial role in maintaining the homeostasis of the airway microenvironment and effectively clearing invading pathogens.

## 3. Airway Cilia in Coronaviral Infections

### 3.1. The Impact of Coronaviruses on Cilia within the Upper Respiratory Tract

Ciliated epithelial cells in the upper respiratory tract represent the primary site of initial coronavirus infection. A number of respiratory viruses, including coronaviruses, influenza viruses, parainfluenza viruses (PIVs), rhinoviruses (RVs), and respiratory syncytial viruses (RSVs) primarily infect ciliated epithelial cells of the respiratory tract [44,45,46,47]. The cilia of the nasal airway epithelium are primarily responsible for cleaning and moistening the surrounding environment [48], whereas the cilia of the olfactory epithelium extend to the dendrites of mature olfactory neurons, which are responsible for odor detection [49]. Single-cell RNA sequencing analysis has demonstrated that the microvilli of respiratory epithelial cilia and sustentacular cells in the olfactory epithelium express high levels of receptors for binding to coronaviruses, including ACE2 and TMPRSS2 [50]. Autopsy samples of the respiratory tract, olfactory mucosa, and whole olfactory bulb taken immediately after death from patients with confirmed cases of SARS-CoV-2 infection demonstrated that cilia and microvilli of sustentacular cells were the primary target cell types infected by the virus [18].

In the nasal epithelia, the SARS-CoV-2 virus binds to the ACE2 receptor, which is located on the surface of the cilia, thereby enabling the virus to traverse the ciliary mucin layer. Subsequently, the virus attaches to the microvilli of the respiratory tract, stimulating their formation, and then returns through the microvilli to the mucus layer. Following that, the virus is dispersed throughout the airway tissue by ciliary beating [43]. In respiratory and olfactory epithelial cells, the low expression of ACE2 necessitates the involvement of neuropilin 1 (NRP1), which is highly expressed on the cilia of olfactory epithelium and olfactory neural progenitor cells, to enhance SARS-CoV-2 binding to the host [51,52]. Thus, viral particles within the nasal cavity exhibit a distinctive affinity for cilia and undergo substantial replication within ciliated cells. Upon entering ciliated cells, the coronavirus causes the cilia to retract into the cell body, resulting in the loss of cilia and the presence of only microvilli [53]. In an investigation of the effect of the coronavirus on human nasal CECs, cilia loss and an abnormal ciliary microtubule structure were observed in 15 healthy patients after three days of nasal administration of HCoV-229E [54]. It can therefore be concluded that cilia in the upper respiratory tract provide a means of entry and replication for the coronavirus and that damage to these cilia may result from viral invasion [43,51,52,53,54].

Infection with coronaviruses in the upper respiratory tract typically results in the subsequent presentation of respiratory symptoms that are similar to those observed in instances of damage to the respiratory cilia in the upper respiratory tract. Infection with a coronavirus may result in the onset of anosmia, or the loss of the sense of smell. A study was conducted to assess the upper respiratory symptoms in 10,415 patients infected with coronaviruses, including 9263 cases of SARS-CoV-2, 461 of SARS-CoV, 283 of MERS-CoV, and 408 of other coronaviruses. The data indicated that for chemosensory dysfunction, loss of smell and taste were reported in 49.6% and 47.9% of cases, respectively. In addition, in the context of sinonasal dysfunction, the presence of runny nose/rhinorrhea/rhinitis and nasal congestion/obstruction/blockage was observed in 10.7% and 8.5% of cases, respectively. It is noteworthy that only patients infected with SARS-CoV-2 exhibited symptoms of loss of smell and taste, whereas the prevalence of sinus dysfunction was comparatively lower in patients with other coronavirus infections [55]. Epidemiological data indicate that loss of smell and taste is the most prevalent symptom among patients infected with SARS-CoV-2 and is the most reliable indicator of infection with the virus [56,57].

The nasal cavity is a crucial gateway for the invasion of SARS-CoV-2. The co-expression of the cell binding factors ACE2 and TMPRSS2 has been observed in the supporting cells (including sustentacular cells, Bowman’s glands, and microvillar cells) and ciliated cells of the olfactory epithelium [58,59]. It has been demonstrated that approximately 47.85% of patients diagnosed with SARS-CoV-2 infection experience olfactory dysfunction, which may persist even after recovery [60,61]. The etiology of acute olfactory loss is multifactorial, encompassing transient alterations in gene expression in olfactory sensory neurons, loss of OSN cilia and receptor downregulation, swelling of the ciliated epithelium, increased mucus production, and inflammation [62,63,64]. In the olfactory system, olfactory cilia extend from olfactory sensory neurons, and olfactory receptors are located on the ciliary membrane [19]. The olfactory cilia exert a direct influence on the function of the olfactory system. Long-term olfactory loss may be attributed to the virus damaging olfactory neurons in the olfactory epithelial cells, which are responsible for detecting odors [65]. However, Mona Khan et al. put forth the proposition that SARS-CoV-2 is not a neurotropic virus and that transient olfactory dysfunction in patients with SARS-CoV-2 infection is caused by transient insufficient support from sustentacular cells in the olfactory epithelium by SARS-CoV-2, which indirectly affects the olfactory sensory neurons [18]. It was not until John B Finlay et al. found that SARS-CoV-2 was absent in the nasopharynx of coronavirus disease 2019 (COVID-19) patients with long-term olfactory loss due to SARS-CoV-2 and that the olfactory loss sustained by these patients was caused by T-cell-mediated inflammation that persists in the olfactory epithelium [66]. A genome-wide association analysis has recently revealed that the odor metabolizing enzyme, a glycosyltransferase encoded by the UDP-glucuronosyltransferase 2A1/2A2 (*UGT2A1*/*2A2*) locus, may play a role as a host factor of SARS-CoV-2 [67]. In particular, the expression of UGT2A1/2A2 in olfactory epithelial cilia provides a genetic link to the biological mechanisms underlying the loss of smell or taste associated with SARS-CoV-2 infection [18].

Secondly, patients infected with coronaviruses may develop pharyngitis. The upper pharynx is composed of ciliated epithelium and lymphoid tissue, which collectively serve to prevent bacterial and viral infections [68]. Nevertheless, the high expression of ACE2 and TMPRSS2 in the upper pharynx provides a conducive environment for the entry and replication of SARS-CoV-2 within the upper pharynx epithelium [69]. If external stimuli cause damage to the cilia in the pharynx or if there is insufficient cilia activity, this can result in an increased secretion of the pharyngeal mucosa and a reduced ability for the mucosa to self-clean. This is favorable for bacteria and viruses to persist and replicate locally, which can lead to infection [70].

### 3.2. The Impact of Coronaviruses on Cilia within the Lower Respiratory Tract

Infection of the lower respiratory tract by SARS-CoV, MERS-CoV, and SARS-CoV-2 in human bronchial epithelial cells (HBECs) is subjective to two regulatory mechanisms: the recruitment of innate immune cells, as exemplified by the upregulation of granulocyte colony-stimulating factor 3 (CSF3), and the disruption of cilia and cytoskeletal structure, including the down-regulation of DNAH7, FOXJ1, and regulatory factor X3 (RFX3) [71]. For instance, the MERS-CoV receptor DPP4 has been identified in bronchial epithelial cilia, and MERS-CoV infection can rapidly induce apoptosis of human bronchial epithelial cells [72]. Moreover, the replication of SARS-CoV-2 infection can result in the rapid loss of the ciliary layer, the loss of the ciliary axoneme, and the mislocalization of the ciliary basal body in the human bronchial epithelial model [5]. It has been demonstrated that four days following inoculation of human airway epithelial cells in culture with bronchoalveolar lavage fluid samples obtained from patients with SARS-CoV-2 infection, a reduction in ciliary movement and the onset of cell pathology can be observed [73]. Similar observations have been made in the lungs of SARS victims, including the loss of cilia, diffuse alveolar damage, haemophagocytosis, bronchial epithelial exfoliation, and squamous metaplasia [74]. Consequently, infection with coronaviruses results in the loss or dysfunction of bronchial cilia, which impairs mucociliary clearance, facilitates viral spread in the airways, and increases the risk of secondary infection in patients.

Ciliary damage in the lower respiratory tract represents the initiating factor for impaired MCC function, which in turn gives rise to bronchitis and pneumonia. In some cases, this can result in the development of severe conditions, including respiratory failure [75]. This symptom is strikingly similar to the lower respiratory symptoms associated with a coronavirus infection. Cough is one of the most conspicuous symptoms of bronchitis and pneumonia, with 67.8% of patients with COVID-19 in China exhibiting overt coughing [76]. It has been demonstrated that SARS-CoV-2 is capable of inducing neuroinflammation and neuroimmune responses through the production of a variety of inflammatory factors, neuropeptides, and other cellular or chemical factors, which activate sensory neurons and result in the release of substance P and neurokinin A. While these neuropeptides may serve to reduce inflammatory responses by activating the vagus nerve, a direct recruitment and activation of immune cells to increase neural tissue and lung inflammation may occur concomitantly, leading to excessive inflammation and ciliary damage [77,78,79]. Subsequently, MCC dysfunction due to ciliary dyskinesia, aberrant ciliary length, and cytoplasmic blebs can precipitate excessive mucus secretion and persistent coughing [80].

Secondly, the severe symptoms triggered by coronavirus infection are mainly manifested as pneumonia, which may subsequently develop into acute lung damage and more severe acute respiratory distress syndrome (ARDS) [81]. When coronaviruses attack the respiratory system, an acute inflammatory response is induced and the airway mucosa and tissues are damaged, leading to bronchial epithelial cell dysfunction, MCC damage, and fibrosis during repair, increasing the risk of bacterial and other viral respiratory infections. These secondary infections are important complications in the rehabilitation process of COVID-19 patients and can cause or exacerbate chronic bronchitis [82]. In addition, bronchiectasis is a long-term consequence of SARS-COVID-19 pneumonia, while ciliary dyskinesia is the main promoter of bronchiectasis [83,84]. This may provide a potential explanation for the frequent association of coronaviruses with refractory or persistent pneumonia.

### 3.3. The Impact of Coronaviruses on Pathogenic Genes of Respiratory Cilia

The occurrence of mutations in genes that are involved in the movement, structure, assembly, and function of ciliary axonemes will result in the development of primary ciliary dyskinesia. In recent years, the identification of nearly 50 cilia-related genes has facilitated the clarification of the genotypic-phenotypic differences associated with PCD diagnosis. The most commonly identified genes are dynein axonemal intermediate chain 1/2 (*DNAI1*/*2*), dynein axonemal heavy chain 5/7 (*DNAH5*/*7*), outer dynein arm docking complex subunit 2 (*ODAD2*), and coiled-coil domain containing 103/114/151 (*CCDC103/114/151*) (outer dynein arms). Other genes include N-DRC, leucine-rich repeat containing 56 (*LRRC56*), coiled-coil domain containing 39/40/65/164 (*CCDC39*/*40*/*65*/*164*), dynein axonemal assembly factor 1/2 (*DNAAF1*/*2*) (dynein arm assembly and docking); radial spoke head component 1/3/4A/9 (*RSPH1*/*3*/*4A*/*9*), dnaJ heat shock protein family (Hsp40) member B13 (*DNAJB13*), and serine/threonine kinase 36 (*STK36*) (central pair and radial spokes). Mutations in these genes can lead to disrupted ciliary beating or totally immotile cilia [85].

Previous investigations have demonstrated that coronaviruses are capable of modulating the expression of genes related to respiratory cilia, which can result in aberrant cilia structure, MCC dysfunction, and ultimately respiratory disease. Following infection of the bronchial epithelium with SARS-CoV-2, a reduction in the expression of cilia regulatory factors, including FOXJ1, RFX3, and DNAH7, was observed. This resulted in the loss of ciliary axonemes, misorientation of basal bodies, and loss of cilia [5]. For example, evidence has been presented indicating that SARS-CoV-2 open reading frame 10 (ORF10) has the capacity to enhance the activity of CUL2^ZYG11B^, which in turn results in the ubiquitination of the ciliary anterograde transport protein named IFT46. This, in turn, has been shown to impair the biogenesis and maintenance of cilia [86]. Following the induction of an inflammatory response by SARS-CoV-2, the expression of transmembrane MUC5AC around cilia is activated, while the activity of the MCC and mucus conversion is inhibited, resulting in ARDS [87]. Conversely, although PCD is characterized by impaired mucociliary clearance and recurrent respiratory infections and failure, a study showed that ALI cultures from PCD patients had fewer SARS-CoV-2-infected cells than healthy donors at 48 h post-infection. This is because PCD patients have low MCT, which reduces the risk of coronavirus infection [43]. In addition, PCD patients take greater precautions to protect themselves, including vaccination, a reduction in social contacts, and the strict use of face masks during pandemics. This may explain why, despite impaired mucociliary clearance, people with PCD are not at increased risk of infection or severe COVID-19 [88].

Single-cell sequencing data of leukocytes and epithelial cells in bronchoalveolar lavage fluid from three patients with severe SARS-CoV-2 infection revealed a significant downregulation of cilia regulatory factors, including *FOXJ1* and *RFX3*, following viral infection. In spite of the aforementioned observations, the expression of cilia-specific kinase NIMA-related kinase 10 (*NEK10*) was found to be significantly down-regulated, while the expression of ATP synthesis-related genes, including ATP synthase F1 subunit epsilon (*ATP5F1E*), ATP synthase membrane subunit C locus 2 (*ATP5MC2*), and ATP synthase membrane subunit G (*ATP5MG*) were found to be dramatically reduced, while the expression of cilia structure genes *DNAI2*, *DNAAF1*, abelson helper integration site 1 (*AHI1*), and *IFT27* was remarkably hindered [6]. In addition to exerting a direct influence on the expression of genes implicated in ciliary dysfunction, viruses may also contribute to cilia deformation, which is associated with the cell cycle. It has been shown that following SARS-CoV-2 infection, the activity of the cell cycle protein cyclin-dependent kinase 1 (CDK1) is significantly diminished, resulting in a blockade at the S/G2 phase. Nevertheless, it remains to be established whether SARS-CoV-2 infection affects cilia development through the cell cycle [89].

Collectively, coronavirus infection may directly or indirectly affect the expression of cilia-related genes, thereby affecting the structure and function of cilia, which in turn may result in an abnormal clearance function of the respiratory tract. Further verifications are required to elucidate the specific regulatory mechanisms and effects of the coronavirus on pathogenic genes associated with cilia.

### 3.4. The Impact of Susceptible Genes for Coronaviruses on Respiratory Cilia

Epidemiological studies have shown that, in recent years, patients infected with SARS-CoV-2 have exhibited a spectrum of symptoms, including the emergence of asymptomatic infections. In order to elucidate the contribution of genetic factors to the risk of SARS-CoV-2 infection and the severity of associated symptoms, scientists have launched the international project “COVID-19 Host Genetics Initiative”. To date, the project has identified 51 sites associated with COVID-19 susceptibility, hospitalization, and severity [90]. The relevant sites of COVID-19 susceptibility/symptom severity are distributed across three main biological pathways: virus entry, respiratory mucus resistance, and type I interferon (IFN-I) [91]. All of these pathways are associated with cilia. A summary of the virus susceptibility genes related to cilia is provided in Table 1.

Firstly, susceptibility genes involved in viral entry include (1) ACE2 and TMPRSS2, which are located on respiratory cilia; (2) signaling threshold regulating transmembrane adaptor 1 (SIT1) encoded by solute carrier family 6 member 20 (*SLC6A20*), which functionally interacts with ACE2; and (3) N-ethylmaleimide sensitive factor (NSF), which mediates vesicular trafficking under the control of both viruses and cilia [92,93,94,95]. Secondly, susceptibility genes associated with airway mucus resistance have been shown to include (1) the MUC family (MUC1, MUC4, MUC16, and MUC5B), which form the primary components of airway mucus and clear pathogens through MCC; (2) Leucine zipper transcription factor like 1 (LZTFL1), a ciliated protein that impairs ciliogenesis by reducing the clearance rate of respiratory viruses during SARS-CoV infection; and (3) transmembrane inner ear (*TMIE*), a cilia-associated candidate gene encoding an inner ear transmembrane protein that affects hearing, olfaction, and protein and vesicle trafficking [87,96,97,98]. Thirdly, type I interferon-related susceptibility genes, such as the C-C motif chemokine receptor (CCR), have been shown to promote an inflammatory response and lead to the abnormal clearance of airway mucus and cilia [99,100,101,102,103,104].

### 3.5. Effects of Respiratory Cilia on the Life Cycle of Coronaviruses

The life cycle of coronaviruses can be divided into the following stages: attachment, entry, replication, assembly, and release. Each of these stages is highly dependent on the host cell. A more comprehensive grasp of the role of airway cilia in the life cycle of coronaviruses will facilitate the implementation of effective intervention strategies.

For a coronavirus to establish an infection in a host organism, it is necessary for the viral genome to be transferred into host cells. The first stage of this process requires the virus to bind to receptors on the host cell membrane. This binding not only enables viral entry into the host cell but also drives the process of membrane fusion. The entry of SARS-CoV, MERS-CoV, and SARS-CoV-2 is facilitated by the S protein, which requires two S protein cleavage events. The first cleavage associated with the enzyme furin occurs at the junction of the S1 and S2 subunits, while the second cleavage event, dependent on TMPRSS2, occurs at the S2 site within the S2 subunit [105]. Following cleavage of the S protein, the S1 subunit of SARS-CoV binds to the ACE2 receptor, with the S2 subunit facilitating fusion of the viral and host cell membranes [106,107]. In the case of MERS-CoV, the S1 subunit binds to DPP4 [108], whereas in the case of SARS-CoV and SARS-CoV-2, the S1 subunit binds to ACE2, with a higher affinity observed for SARS-CoV.

A substantial number of specific receptors have been identified on the ciliary membrane, including ACE2, TMPRSS2, NRP1, and DPP4. These receptors enable the cilia to detect, transduce, and translate external mechanical or chemical stimuli within the cells [109,110]. The ACE2 receptor is robustly localized within the motile cilia of airway epithelial cells, extending from the nasal cavity down to the lower bronchus. This provides a large surface area for SARS-CoV-2 binding and cellular entry [92]. TMPRSS2 is highly expressed in the nasal cavity and airways and is stably localized to the cilia [4]. NRP1 is highly expressed in the olfactory epithelial cells of patients with COVID-19 and is situated in close proximity to the olfactory cilia [51]. DPP4 was identified as being expressed in human bronchial, alveolar epithelial cells and macrophages. A reduction in cilia was observed following MERS-CoV infection, which was accompanied by a decline in DPP4 levels [111]. It is also noteworthy that the differential localization of virus-binding receptors in the airways results in differential susceptibility to infection. For example, the primary targets of SARS-CoV and MERS-CoV are the lower respiratory tract, with less nasopharyngeal infection. This is evidenced by the absence of such symptoms in MERS patients [112,113]. Following infection of the OE with SARS-CoV-2, damage or loss of cilia, along with down-regulation of the expression of the ciliary olfactory receptor and key olfactory signaling factors cyclic nucleotide gated channel subunit alpha 2 (CNGA2) and ADCY3, were observed in mice, resulting in abnormal olfaction in mice [114]. It can thus be postulated that these cilium-binding receptors may exert a role in determining the influence on the tissue tropism of the coronavirus.

Once the coronavirus has bound to receptors on the surface of host cells, it gains entry through two pathways: direct fusion with or penetration of the cell membrane and endocytosis [115]. Previous studies have shown that SARS-CoV and MERS-CoV predominantly enter host cells by endocytosis, with SARS-CoV fusing with late endosomes and MERS-CoV fusing with early endosomes [116]. However, the entry of SARS-CoV-2 is dependent on both routes. In the process of viral fusion, upon binding of SARS-CoV-2 to ACE2, the viral S protein was activated by TMPRSS2 for ciliary membrane fusion at the ciliary surface. Subsequently, the viral RNA genome is released into the ciliary matrix and transported from the cilium to the cytosol by ciliary dynein. With regard to the endocytic pathway, it has been demonstrated that, following the binding of SARS-CoV-2 to ACE2, the virus is transported from the tip of the cilium to the cell body by ciliary dynein, where it subsequently fuses with the lysosome [43]. Similarly, SARS-CoV enters host cells through an ACE2-mediated endocytic pathway that is independent of clathrin and caveolae [117]. With regard to cilia, a membrane domain at the base of some motile cilia is designated the “ciliary pocket”, may function as an endocytic platform for cilia-associated vesicular trafficking, thereby participating in viral endocytosis [118]. In addition, the autophagy-linked plasma and placenta associated 8 (PLAC8), a key host factor for SARS-CoV-2 entry into human cells, is highly expressed in ciliated respiratory cells, which can regulate autophagic lysosomal compartments and influence the intracellular fate of endocytic viral particles [119]. Thus, cilia can mediate viral endocytosis, allowing the virus to be transported to endosomes, which ultimately fuse with lysosomes and/or are engulfed by autophagosomes [120].

Upon entering a host cell, coronaviruses begin to replicate rapidly, leading to the apoptosis of massive epithelial and endothelial cells and then triggering a significant inflammatory response. In the early stages of coronavirus infection, ciliated cells show multiple immune responses and are most permissive for viral replication [121]. However, prolonged or excessive inflammatory responses can lead to damage or loss of cilia, thereby impairing normal ciliary function and the ability to clear mucus and foreign bodies from the airways. This can create an environment conducive to the exacerbation of lung disease [122]. For example, significant pro-inflammatory responses, commonly known as cytokine release syndrome (CRS), have been reported in patients infected with SARS-CoV, MERS-CoV, and SARS-CoV-2 [123]. In addition, excessive activation of peripheral cluster of differentiation 4 receptors (CD4) and cluster of differentiation 8 receptors (CD8) T cells characterized by high levels of CCR6 + TH17 has been observed in patients with COVID-19. This is associated with a hyperinflammatory response [124]. Similarly, elevated levels of TH17 and activation of Interleukin-17 (IL-17)-related pathways have been reported in individuals infected with SARS-CoV and MERS-CoV [125,126]. Both CRS and T-cell dysregulation have been linked to the development of interstitial pneumonia and ARDS, which in turn disrupt the normal function of airway cilia, providing a basis for the development of coronavirus-induced ARDS [127].

Extracellular vesicles (EVs) are a type of vesicle with a bilayer membrane structure that are secreted by cells into the extracellular environment. They contain lipids, proteins, and nucleic acids that are essential for remote signaling [128]. It has been demonstrated that ciliated membranes are capable of releasing extracellular vesicles through budding, with this particular source of microcapsules being designated as ciliated exosomes [129]. Following a 24 h infection of nasal epithelial cells with coronaviruses, small viral vesicles accumulate in close proximity to or within the microvilli, while larger vesicles are located in the cytoplasm in the vicinity of the cell [43]. Cilia therefore serve as a bridge for material transport and signaling between cells, both by receiving the virus within the cell and by releasing the virus outside the cell [130].

It can thus be concluded that the interaction between coronaviruses and airway cilia is a complex process, influenced by factors such as the life cycle of the virus and the immune status of the host. A more profound comprehension of the relationship between coronaviruses and airway cilia will facilitate a better grasp of the mechanism underlying coronavirus infection, thereby paving the way for more effective strategies for the prevention and treatment of coronavirus infection.

## 4. Therapeutic Measure

### 4.1. Targeting Viral Infection as Therapeutics

Over the past decade, three major outbreaks of coronaviruses have been documented. The first of these was SARS-CoV in 2002, followed by MERS-CoV in 2012 and then SARS-CoV-2 in 2019. Notwithstanding the advances in health infrastructure and the knowledge to control infectious diseases, the emergence and re-emergence of coronavirus pandemics may be precipitated by environmental and climate change. In order to be adequately prepared for the next global virus outbreak, it is imperative that broad-spectrum antivirals against highly pathogenic coronaviruses are developed. Despite the structural diversity of coronaviruses, the viral life cycle exhibits a number of common features, including entry, replication, assembly, and release. The design of antiviral drugs based on the different segments of the viral life cycle has yielded notable therapeutic outcomes (Table 2).

Firstly, to prevent viral entry, antivirals have been designed to target host viral receptors, including ACE2, TMPRSS2, NRP1, and DPP4. Drugs currently in development include ACE2 inhibitors, such as human recombinant soluble ACE2 (hrsACE2), which competitively binds to the S protein, thereby reducing damage to the lungs, kidneys, and heart [131]. Inhibitors of TMPRSS2, such as the serine protease inhibitor camostat mesylate (Ono Pharmaceutical, Osaka, Japan), have been demonstrated to impede the activity of TMPRSS2, thereby blocking the infection of lung cells by SARS-CoV-2 [132]. Pruxelutamide (Kintor, Suzhou, China), an androgen receptor antagonist, has been demonstrated to reduce the expression of ACE2 and TMPRSS2 and to inhibit the production of inflammatory cytokines. This consequently prevents the onset of cytokine storms [133]. Inhibitors of NRP1, such as EG 00229 trifluoroacetate (Adooq Bioscience, Irvine, CA, USA), are small-molecule antagonists that have been shown to inhibit the binding of NRP1 to the S protein and diminish infectivity in vitro [134]. With regard to the DPP4 inhibitor, the amino acid residues responsible for binding DPP4 to SARS-CoV-2 were found to be identical to those that facilitate binding of DPP4 to MERS-CoV. Further investigation is required to ascertain the potential role of DPP4 inhibitors in the treatment of patients with COVID-19 [135]. Subsequent to the binding of coronaviruses to receptors, coronaviruses enter the cell by endocytosis. During this process, compounds such as apilimod (LAM Therapeutics, Livermore, CA, USA) and colchicine have been demonstrated to prevent viral entry by interfering with the transport and maturation of endosomes to lysosomes [136]. Chloroquine (CQ, Gilead Sciences, Foster City, CA, USA) and hydroxychloroquine (HCQ, Sanofi, Paris, French) are antimalarial drugs that have been demonstrated to increase the pH of lysosomes and trans-Golgi network vesicles, thereby inhibiting viral invasion [137].

Secondly, a number of enzymes are involved in the synthesis of viral RNA, including the RNA-dependent RNA polymerase (RdRp), the 3-chymotrypsin-like protease (3CLpro), the papain-like Protease (PLpro) and the viral helicase. Drugs developed against these enzymes include RdRp inhibitors, such as remdesivir (Gilead Sciences), sofosbuvir (Gilead Sciences), and azvudine (Genuine Biotech, Pingdingshan, China). The inhibition of viral replication is achieved by blocking the viral RNA-dependent RNA polymerase, which results in the cessation of the process of reverse transcription [138,139,140]. Inhibitors of 3CLpro, such as nirmatrelvir/ritonavir (Paxlovid, Pfizer, New York, NY, USA), have been demonstrated to effectively inhibit the replication of coronaviruses [141]. HL-21, a small molecule inhibitor of PLpro, has been shown to exhibit potent inhibitory activity against a number of coronaviruses [142]. Inhibitors of the helicase enzyme, such as SSYA10-001, aryl diketone acid (ADK), and dihydroxychromone, have the potential to disrupt the function of non-structural protein 13 (NSP13), which may result in the restriction of viral replication [143].

The final stage of the viral life cycle is viral assembly, which occurs after the complete viral genome has been translated into structural proteins. Inhibitors that target structural proteins can also exert an antiviral effect at this stage. Inhibitors of the E protein, including hexamethylene amiloride (HMA) and amantadine (AMT), as well as their combinations, have been identified as potential antiviral agents [144]. The N protein inhibitors PJ34 and 5-benzyloxylamine [145] and the S protein-neutralizing antibody 2G1 have been shown to exhibit antiviral properties [146].

### 4.2. Promoting Cilia Recovery as Therapeutics

In view of the fact that damage to the airway cilia caused by the coronavirus results in MCC dysfunction, there is a clear requirement for the development of drugs that enhance MCC and improve lung function (Table 2). For example, Bronchitol^®^ (Pharmaxis Ltd., Sydney, Australia) is an inhaled mannitol dry powder preparation that has been demonstrated to enhance mucociliary clearance function by increasing the osmotic pressure of airway surface fluid [147]. The β-adrenergic agonists and cholinoids (such as acetylcholine and pilocarpine) can increase the frequency of airway ciliary beating and improve the efficiency of mucociliary clearance [148,149]. It has been observed that terbutaline (Sanofi) affects Cl^−^ transport in the airway epithelium, thereby improving MCC [150]. Amiloride (Merck, Darmstadt, Germany), a Na^+^ channel blocker, has been demonstrated to improve the viscosity of sputum and facilitate mucus clearance over an extended period, thereby protecting the airways from intraluminal obstruction and improving airflow [151]. The β-agonists such as salmeterol (GSK, London, UK) and salbutamol (GSK), have been shown to elevate intracellular cyclic adenosine monophosphate (cAMP) levels, which in turn enhances the frequency of ciliary beating [152].

In addition to viral pathogenicity, ciliary dysfunction caused by the inflammatory response constitutes a significant contributory factor in the development of ARDS. It is therefore of the utmost importance to control cytokine production and the inflammatory response in cases of COVID-19. A number of immunomodulatory drugs have shown considerable therapeutic efficacy in the treatment of severe cases of the virus. For example, glucocorticoids such as dexamethasone (Merck), hydrocortisone (Merck), prednisolone (Merck), and methylprednisolone (Upjohn, Cecil Township, PA, USA) have anti-inflammatory and immunomodulatory effects. However, the timing and dose of treatment must be considered during application [153]. Janus kinase inhibitors, such as baricitinib (Incyte, Wilmington, DE, USA), fostamatinib (RIGL, South San Francisco, CA, USA), nezulcitinib (TBPH, South San Francisco, CA, USA), ruxolitinib (Novartis, Basel, Switzerland), and pacritinib (CTI BioPharma, Seattle, WA, USA), have been demonstrated to reduce inflammation by inhibiting the JAK-STAT pathway [154]. Interleukin receptor antagonists, including tocilizumab (CHGCY, Tokyo, Japan), sarilumab (Sanofi), and anakinra (Sobi, Stockholm, Sweden) have been demonstrated to reduce the inflammatory response by blocking pro-inflammatory cytokines such as IL-6 and IL-1 [155]. Interferons, such as IFN-α and IFN-β, are crucial in the antiviral immune response to viral infections [156]. Non-steroidal anti-inflammatory drugs, such as topotecan (GSK) and vilobelimab (InflaRx, Jena, Germany), have been shown to provide relief from hyperinflammatory symptoms and to prevent their progression [157].

**Table 2:** Therapeutic measures.

Viral Life Cycle	Potential Therapeutics	Direct and Indirection Associations with Cilia during Infection	References
Virus ad-sorption Virus ad-sorption	1 Human Recombinant Soluble ACE2	ACE2 competes with S protein, thereby reducing the damage caused by the coronavirus to organs such as the lungs, kidneys, and heart.	[131]
	2 Camostat Mesilate	Inhibiting the activity of TMPRSS2 can block the infection of lung cells by SARS-CoV-2.	[132]
	3 Pruxelutamide	Lowering the expression of ACE2 and TMPRSS2, while suppressing the production of inflammatory cytokines through activation of the NRF2 pathway.	[133]
	4 EG 00229 trifluoroacetate	Inhibition of NRP1-S protein binding and reduction in virus infectivity.	[134]
Virus entry	1 Apilimod/Colchicine	Impacting the trafficking and maturation of lysosomes, thereby preventing the entry of viruses.	[136]
	2 Chloroquine (CQ)/Hydr-oxychloroquine (HCQ)	Increasing the pH of lysosomes and trans-Golgi network vesicles, thereby inhibiting virus assembly.	[137]
Virus synthesis	1 Remdesivir/Sofosbuvir/Azvudine	Inhibition of the virus RdRP leads to termination of the virus during the reverse transcription process, thereby inhibiting virus replication.	[138,139,140]
	2 Paxlovid	A potent inhibitor of 3CLpro can effectively inhibit the in vitro activity of coronavirus.	[142]
	3 SSYA10-001/ADK/5,7-Dihydroxychromone	Inactivating NSP13 thereby inhibiting virus replication.	[143]
Virus assembly	1 PJ34/5-benzyloxygramine	N protein inhibitor.	[144]
	2 Hexamethylene amiloride (HMA)/Amantadine (AMT)	E protein inhibitor.	[145]
	3 2G1	S protein neutralizing antibody.	[146]
	4 Glucocorticoid	Having anti-inflammatory and immunomodulatory effects.	[153]
Cilium recovery	1 Bronchitol^®^ (Pharmaxis Ltd.)	By increasing the permeability of bile, enhancing the mucociliary clearance function.	[157]
Cilium recovery	2 β-adrenergic receptor agonists	Increasing the frequency of ciliary beats in the respiratory tract to improve the efficiency of mucociliary clearance.	[148]
	3 Terbutaline	Influencing the transport of Cl^−^ in the airway epithelium to enhance mucociliary clearance.	[150]
	4 Amiloride	Long-term inhalation can improve the viscosity of sputum and mucociliary clearance, protecting the respiratory tract from intrapulmonary obstruction and improving airflow.	[151]
	5 Acetylcholine/Pilocarpine	Increase in ciliary beat frequency.	[149]
	6 Baricitinib/Fostamatinib/Nezulcitinib/Ruxolitinib/Pacritinib	Inhibition of JAK-STAT signaling pathway to alleviate inflammation.	[154]
	7 Tocilizumab/Sarilumab /Anakinra	Blocking pro-inflammatory cytokines such as IL-6 and IL-1 to alleviate the inflammatory response.	[155]
	8 IFN-α/IFN-β	Play a crucial role in the antiviral immune response against viral infections.	[156]
	9 Topotecan/Vilobelimab	It can alleviate super-inflammatory symptoms to prevent them from becoming worse.	[157]

Abbreviations: 3CLpro, 3-chymotrypsin like protease; ACE2, angiotensin converting enzyme 2; AMT, amantadine; CQ, chloroquine; E, envelope; HCQ, hydroxychloroquine; HMA, hexamethylene amiloride; IL, interleukin; IFN, interferon; NRF2, nuclear respiratory factor 2; NRP1, neuropilin 1; N, nucleocapsids; NSP13, non-structural protein 13; RdRp, RNA-dependent RNA polymerase; S, spike; TMPRSS2, transmembrane protease serine 2.

## 5. Conclusions

In summary, airway cilia play a pivotal role in the regulation of coronaviruses, which are involved in all stages of the viral life cycle and are controlled by multiple molecular mechanisms. Firstly, respiratory cilia constitute the primary line of defense in the airways, with the function of clearing pathogen-containing mucus through rhythmic beating. Secondly, the ciliary membrane expresses receptors that bind to viruses. Following the invasion of the airways by coronaviruses, specific targeting of cilia ensues, resulting in disruption to all aspects of cilia biology. These include alterations to motility, beat coordination, ultrastructure. and gene expression, which collectively lead to the rapid destruction of the MCC, thereby facilitating further spread. In addition, respiratory cilia are involved in the processing of sensory receptor responses, the regulation of inflammatory pathway signaling, and the monitoring of immune surveillance. Cilia are capable of detecting foreign substances, such as viruses, and initiating an immune response, which in turn triggers the release of inflammatory mediators. This attracts immune cells to the site of infection, where they can clear the infection. Nevertheless, the precise molecular mechanisms underlying the mechanical and signaling sensing functions of cilia, as well as the molecular mechanisms promoting the development of respiratory disease following the loss of these functions due to ciliary damage, remain unclear and require further investigation. It can thus be proposed that the maintenance of respiratory cilia health and functionality represents a significant strategy for the prevention and treatment of coronaviruses. Further elucidation of the molecular mechanisms of coronavirus-induced ciliary damage may facilitate the development of novel treatment options for respiratory infections. The appropriate pharmacological targeting of respiratory cilia, such as the enhancement of their sensing function to increase their mechanical clearance ability, may improve the clearance of viruses and prevent their entry into cells.

In addition, in the context of the relationship between upper and lower respiratory cilia and coronaviruses, it has been established that the nasal cavity contains two distinct types of cilia: respiratory cilia and olfactory cilia (Figure 1). The primary function of the respiratory cilia is to assist in the clearance of foreign particles and microorganisms, including dust, bacteria, and viruses. Olfactory cilia are primarily responsible for the detection of odor molecules in the air and are involved in the formation of olfactory perception. Nevertheless, the coronavirus-binding receptors ACE2 and TMPRSS2 are markedly expressed on the cilia of respiratory epithelial cells and the microvilli of olfactory epithelial sustentacular cells, though not specifically localized on olfactory cilia. Thus, various types of cilia may have different roles to play in the process of coronavirus entering the respiratory tract. In addition, following a respiratory infection, the upper and lower respiratory tract cilia move in different directions to transport mucus containing dust and pathogens to the pharynx, where the mucus ball is either swallowed or coughed out to clear the airway. However, the specific regulatory mechanisms and functional differences between upper and lower airway cilia remain unclear. Further studies of respiratory cilia are required to gain insight into the potential heterogeneity of cilia, both between different regions of the respiratory tract and within the same region. This will enable an investigation of whether heterogeneity in respiratory cilia is associated with the symptoms of different respiratory infections caused by coronaviruses and facilitate the development of specific treatments for the symptoms of different respiratory infections.

## Figures and Tables

**Table 1:** Virus susceptibility genes associated with cilia.

Biological Pathway	Viral Susceptibility Gene	Association with Cilia	Region of Action	References
Virus entry	1 *ACE2*	ACE2 is localized within the motor cilia of airway epithelial cells and is the initial site of SARS-CoV-2 entry during host respiratory transmission.	Respiratory epithelial cell	[92]
	2 *TMPRSS2*	Co-localization with ACE2 as a target of SARS-CoV-2 on respiratory cilia.	Respiratory epithelial cell	[93]
	3 *SLC6A20*	Encodes proline transporter protein 1 (SIT1), which functionally interacts with ACE2.	Renal epithelial cell	[94]
	4 *NSF*	Vesicle-fusing ATPase that is required to mediate vesicular transport. Both viruses and cilia are involved in the vesicular transport pathway.	Eukaryotic cell	[95]
MCC	1 *MUC1*/*MUC4*/*MUC16*	MUC1, MUC4, and MUC16 are transmembrane mucins in the respiratory tract, serving as the main components of airway mucus. They can prevent microbial invasion, act as releasable decoy receptors, and facilitate the removal of microorganisms from the respiratory tract through the coordinated beating of cilia.	Airway	[87]
	2 *MUC5B*	The primary structural component of airway mucus, capable of effectively clearing pathogens through mucociliary clearance.	Airway	[87]
	3 *LZTFL1*	A ciliary protein, SARS-CoV may weaken the airway virus clearance rate by regulating LZTFL1 and influencing ciliogenesis	Lung epithelial cells, Hypothalamic neurons	[96,97]
	4 *TMIE*	A candidate gene related to cilia. It encodes a transmembrane inner ear protein that is essential for the development of cochlear sensory cilia. This protein can affect hearing, smell, as well as protein and vesicle transport.	Sensory cilia	[98]
Inflammation response	1 *CCR1*	In patients with PCD, the expression levels of CCR1, CCR2, and CCR5 on monocytes are similar to those in monocytes from healthy individuals; however, upon stimulation by LPS, peptidoglycan, or dsRNA, they produce high levels of pro-inflammatory cytokines and chemokines.	Monocytes from PCD patients	[99]
	2 *CCR2*	In mice with a deficiency of ciliary kinase (LKB1 or PKD1), the renal tubules and bile duct epithelial cells show increased expression of CCL2, which results in the peritubular accumulation of CCR^2+^ monocytes, thereby promoting the ciliopathy phenotype.	Renal tubular epithelial cells, Biliary epithelial cells	[100,101]
	3 *CCR3*	Chronic rhinosinusitis with nasal polyps (CRSwNP) is characterized by type 2 inflammation and increased expression of CCR3, which promotes inflammatory response and abnormal ciliary function.	Nasal polyp mucosa	[102]
	4 *CCR5*	Bronchial epithelial cells express HIV receptors CD4, as well as co-receptors CCR5 and CXCR4, and HIV infection can inhibit bronchial mucociliary clearance.	Bronchial epithelial cell	[103]
	5 *CRHR1*	Expression is reduced in patients with PCD.	Mouse airway epithelial trachea	[104]

Abbreviations: *ACE2*, angiotensin converting enzyme 2; CCR, C-C motif chemokine receptor; CCL2, C-C motif chemokine ligand 2; CRSwNP, chronic rhinosinusitis with nasal polyps; CD4, cluster of differentiation 4 receptors; CXCR4, C-X-C chemokine receptor type 4; *CRHR1*, corticotropin releasing hormone receptor 1; dsRNA, double-stranded RNA; HIV, human immunodeficiency virus; LPS, lipopolysaccharides; LKB1, liver kinase B1; *LZTFL1*, leucine zipper transcription factor like 1; MUC, mucin; *NSF*, N-ethylmaleimide sensitive factor; PCD, primary ciliary dyskinesia; PKD1, Polycystin 1; *SLC6A20*, solute carrier family 6 member 20; SIT1, signaling threshold regulating transmembrane adaptor 1; *TMPRSS2*, transmembrane protease serine 2; *TMIE*, transmembrane inner ear.

## Data Availability

Not applicable.

## Abbreviations

ACE2, angiotensin converting enzyme 2; ARDS, acute respiratory distress syndrome; ATP, Adenosine triphosphate; ATP5F1E, ATP synthase F1 subunit epsilon; ATP5MC2, ATP synthase membrane subunit C locus 2; ATP5MG, ATP synthase membrane subunit G; AHI1, Abelson helper integration site 1; ADCY3, adenylate cyclase 3; ADK, aryl diketone acid; AMT, amantadine; ALI, air-liquid interface; BPIFA1, BPI fold containing family A member 1; CoVs, coronaviruses; CECs, ciliated epithelial cells; 3CLpro, 3-chymotrypsin like protease; COVID-19, coronavirus disease 2019; CSF3, colony-stimulating factor 3; CCDC, coiled-coil domain containing; CDK1, cyclin-dependent kinase 1; CCR, C-C motif chemokine receptor; CNGA2, cyclic nucleotide gated channel subunit alpha 2; CRS, cytokine release syndrome; CD, cluster of differentiation; CQ, chloroquine; cAMP, cyclic adenosine monophosphate; CCL2, C-C motif chemokine ligand 2; CRSwNP, chronic rhinosinusitis with nasal polyps; CXCR4, C-X-C chemokine receptor type 4; CRHR1, corticotropin releasing hormone receptor 1; DPP4, dipeptidyl peptidase 4; DNAH, dynein axonemal heavy chain; DNAI, dynein axonemal intermediate chain; DNAAF, dynein axonemal assembly factor; DNAJB13, dnaJ heat shock protein family (Hsp40) member B13; dsRNA, double-stranded RNA; EPCAM, epithelial cell adhesion molecule; EVs, extracellular vesicles; E, envelope; FOXJ1, forkhead box protein J 1; GBCs, globose basal cells; HBECs, human bronchial epithelial cells; HBCs, horizontal basal cells; HCQ, hydroxychloroquine; HMA, hexamethylene amiloride; HIV, human immunodeficiency virus; hrsACE2, human recombinant soluble ACE2; ICAM1, intercellular adhesion molecule 1; IFT, intraflagellar transport; IFN, interferon; IL, Interleukin; KRT, keratin; LRRC56, leucine-rich repeat containing 56; LPS, lipopolysaccharides; LKB1, liver kinase B1; LZTFL1, leucine zipper transcription factor like 1; MCC, mucociliary clearance; MCT, mucociliary transport; MASH1, mammalian achaete-scute homologue 1; MUC, mucin; N-DRC, nexin-dynein regulatory complex; NRF2, nuclear respiratory factor 2; NRP1, neuropilin 1; N, nucleocapsids; NEK10, NIMA related kinase 10; NSF, N-ethylmaleimide sensitive factor; NSP13, non-structural protein 13; OE, olfactory epithelium; OSN, olfactory sensory neurons; OMP, olfactory marker protein; ODAD2, outer dynein arm docking complex subunit 2; ORF10, open reading frame 10; PCL, periciliary layer; PAX6, paired box 6; PIVs, parainfluenza viruses; PCD, primary ciliary dyskinesia; PLC-β2, phospholipase C-β2; PLAC8, placenta associated 8; PLpro, papain-like Protease; PKD1, Polycystin 1; RBD, receptor-binding domain; RE, respiratory epithelium; RP1, retinitis pigmentosa protein 1; RVs, rhinoviruses; RSVs, respiratory syncytial viruses; RFX3, regulatory factor X3; RSPH, radial spoke head Component; RdRp, RNA-dependent RNA polymerase; S, spike; STK36, serine/threonine kinase 36; SIT1, signaling threshold regulating transmembrane adaptor 1; SPA17, sperm autoantigenic protein 17; SPAG6, sperm associated antigen 6; SOX2, SRY-box transcription factor 2; SLC6A20, solute carrier family 6 member 20; TMPRSS2, transmembrane protease serine 2; T2Rs, bitter taste receptors; TRP, transient receptor potential; TAS2R38, taste 2 receptor member 38; TRPV4, transient receptor potential vanilloid 4; TRPM5, transient receptor potential melastatin 5; TUBB3, tubulin beta 3 class III; TMIE, transmembrane inner ear; UGT, UDP-glucuronosyltransferase.
